# Trial of novel image-enhancement software to assist medical students in identifying dermoscopic features of melanoma

**DOI:** 10.1093/skinhd/vzag063

**Published:** 2026-05-21

**Authors:** Stuart Farmer, Simone Harrison, Jeremy Hudson, Nichole Harvey, Oyelola Adegboye, Clare Heal

**Affiliations:** Skin Institute, Auckland, New Zealand; College of Medicine and Dentistry, James Cook University, Townsville, QLD, Australia; College of Medicine and Dentistry, James Cook University, Townsville, QLD, Australia; College of Medicine and Dentistry, James Cook University, Townsville, QLD, Australia; North Queensland Skin Centre, Townsville, QLD, Australia; College of Medicine and Dentistry, James Cook University, Townsville, QLD, Australia; College of Medicine and Dentistry, James Cook University, Townsville, QLD, Australia; Menzies School of Health Research, Charles Darwin University, Darwin, NT, Australia; College of Medicine and Dentistry, James Cook University, Townsville, QLD, Australia

## Abstract

**Background:**

Early detection of melanoma is central to reducing the disease-related burden. The dermatoscope is used by skin cancer clinicians to help identify features of melanoma in a skin lesion. Features can be subtle and identification can be a challenge for early dermoscopists.

**Objectives:**

To investigate the utility of novel image-enhancement software as a possible aid for beginner dermoscopists in identifying melanoma features. The software had previously been developed for optometry for the diagnosis and monitoring of anterior eye conditions.

**Methods:**

Following an introductory lecture on dermoscopy, third-year medical students at James Cook University were asked to complete a 10-lesion test set of dermoscopic images on benign lesions and cutaneous melanoma (CM). Students scored the presence or absence of some melanoma-related features (asymmetry, blue–grey colour, atypical network) and made a diagnosis for a standard image and then for the matching enhanced image.

**Results:**

In total, 61 students participated. Total performance scores did not differ significantly between standard and enhanced images of the benign lesions. However, students correctly identified CM-related features (blue–grey colour *P* = 0.01, atypical network *P* < 0.001) and diagnosed melanoma correctly (*P* = 0.005) significantly more often when observing the enhanced images of CM compared with standard images. Participants also reported a subjective improvement in diagnostic confidence when using the enhanced images.

**Conclusions:**

The image-enhancement software shows promise in assisting beginner dermoscopists in identifying some features of melanoma and could be tested with more advanced dermoscopists along with a wider range of dermoscopic features. The software could potentially be made widely and cheaply available in the form of a mobile phone application or be incorporated into an existing image-management platform.

## Graphical Abstract

**Graphical Abstract:**
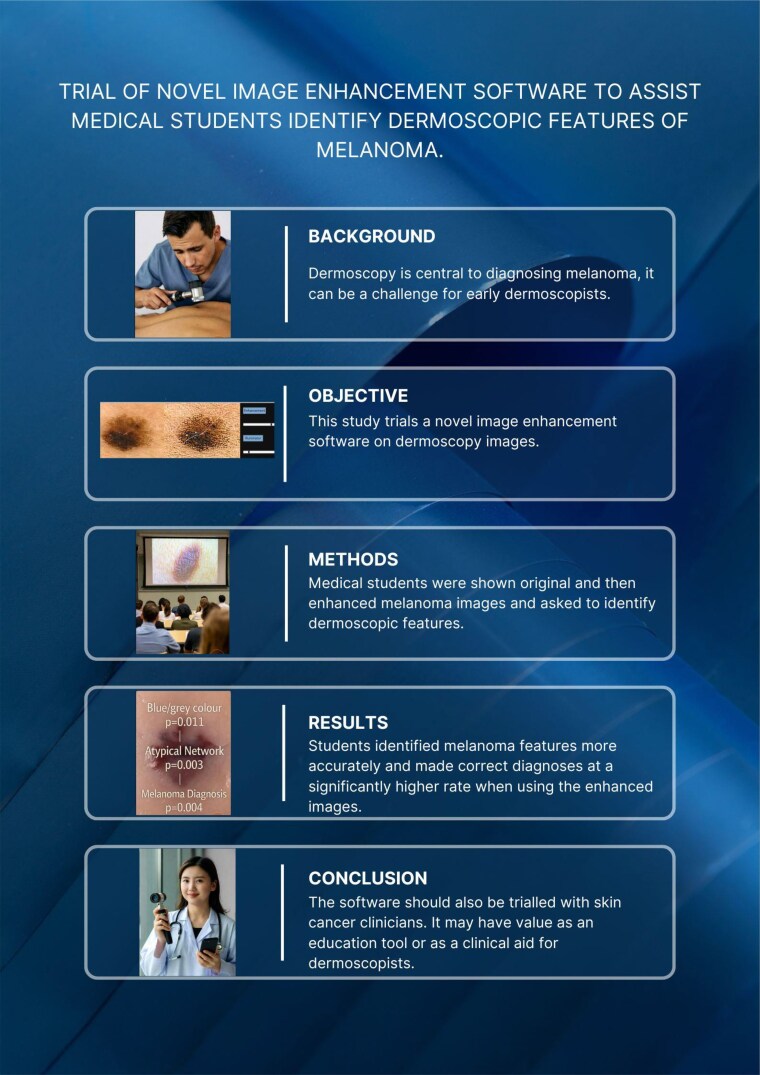

## Teaser

Early detection of melanoma is central to reducing the disease-related burden. Skin cancer clinicians use a dermatoscope to help identify melanoma-related features in a skin lesion. Dermoscopic clues can be subtle and a challenge for early dermoscopists to recognize. This study trialled the application of a novel image enhancement software to dermoscopic images. The software was initially developed for use in optometry. Third-year medical students at James Cook University were asked to complete a test set of images that contained both benign lesions and melanoma. The students identified melanoma-related features and made a diagnosis on original images and then enhanced images. There was no difference in scores for the benign lesions; however, the students identified some melanoma-related features and made a correct diagnosis of melanoma at a significantly higher rate with the enhanced images than with the original images.

What is already known about this topic?

- Early detection of melanoma is central to reducing the disease-related burden.
- The dermatoscope is used by skin cancer clinicians to help identify features of melanoma in a skin lesion.
- Features of early melanoma can be subtle and identification can be a challenge for early dermoscopists.

What does this study add?

- This study assesses the use of a novel image-enhancement software (previously used in optometry) applied to dermoscopic images.
- The study showed that medical students were able to correctly identify some dermoscopic features of melanoma and make a correct diagnosis at a significantly higher rate with the enhanced images than with standard images.
- The software should be trialled with further melanoma features and with actual skin cancer clinicians.
- The software could be made available as an educational tool or incorporated into existing image management systems to aid students or clinicians with the identification of melanoma.

Australasia has the highest incidence of cutaneous melanoma (CM) and CM-related deaths globally.^1^ Early detection is a vital part of reducing this disease burden.^2^ A substantial increase in the number of cases of CM in Australia is expected by 2030. A large proportion of these will be managed in primary care and relevant clinical training will need to be expanded to meet this service need.^3^

Dermoscopy, ‘the stethoscope of the skin’, has been shown to increase the sensitivity of detecting CM by between 9- and 15-fold compared with clinical examination using the naked eye alone.^4^ Current recommendations are that clinicians conducting skin examinations in Australasia should be proficient in dermoscopy, with training commencing early in their medical education.^3^ Recognizing subtle dermoscopic features may be challenging for early-career and experienced dermoscopists, potentially leading to an underdiagnosis of CM.^5,6^

Automated serial total-body photography, with computer assessment of images along with dermoscopy, is an example of recent technological advances that can help identify CM over time.^7,8^ Using multispectral skin analysis or reflectance confocal microscopy in conjunction with dermoscopy has also been shown to improve the sensitivity and specificity for CM diagnosis.^9,10^ However, cost and accessibility are barriers to the widespread use of these expensive systems in primary care or remote settings.

In contrast, some low-cost methods for enhancing dermoscopic images have been identified. A high dynamic range (HDR) setting on mobile phone cameras made it easier to identify dermoscopic features in hypopigmented lesions.^11^ A simple mobile phone editing application (Samsung, Seoul, South Korea) was shown to enhance the appearance of vessels and other dermoscopic structures in a mucocoele.^12^ Feature-amplifying dermoscopy is an example of an image-enhancing algorithm. This technique was used by Blum *et al.* in clinical practice with the FotoFinder dermoscopy system to help evaluate melanocytic skin tumours.^13^ Blum *et al.* published a case report of feature-amplifying dermoscopy and noted that image enhancement was a potentially simple and cost-effective addition to digital dermoscopy.^13^

General image-processing tools use contrast or HDR for image enhancement, but these can saturate the image or result in loss of original data. Sparca Corporation (Fort Lauderdale, FL, USA) has developed a novel algorithm-based signal-enhancing software; this operates at an individual pixel level with no loss of data (https://www.sparca.com). Commercial application of this software to date includes a platform that enables opticians to image and grade anterior eye conditions (https://aos-hub.com). The first author (S.F.) was presented with the opportunity to apply the image-enhancement software to dermoscopy.

S.F. informally presented enhanced dermoscopy images to work colleagues, including intermediate and expert dermoscopists. Feedback from the intermediate dermoscopists – nurse practitioners with 1–5 years of clinical experience – was the most enthusiastic for identifying CM-related features. It was then postulated that early-career dermoscopists would also find the enhancements helpful for identifying CM features. This study looked at the impact of enhanced dermoscopy images on the identification of CM features compared with standard images for novice dermoscopists in the form of third-year medical students at James Cook University, Australia.

## Materials and methods

### Study design

This was an experimental study with a within-participant design. An online test set was created using Google Forms consisting of standard and enhanced dermoscopic images (×10 magnification) for each of 10 flat, ambiguous and at least partially pigmented lesions. The lesions used were either lentigo/atypical melanocytic naevi or melanoma *in situ* (MIS)/early CM and were sourced from the clinical practice of the first author (S.F.) Histology had been obtained for all 10 cases. The seven cases of MIS/early CM were interspersed with the three benign cases. The presence or absence of dermoscopic features in each image was agreed upon by a group of experienced skin cancer clinicians (Skin Cancer College Australasia and NZ Skin Cancer Doctors).

### Study population

The entire cohort of 196 third-year undergraduate medicine students enrolled at James Cook University in Townsville, Australia, in 2023 was sent a group message inviting the voluntary participation of interested students.

Students were taught to use the 3-point checklist to recognize lesion asymmetry, the presence of blue or white colouration, and an atypical pigment network in dermoscopic images during a 50-min compulsory lecture conducted by J.H.^14,15^ Data collection was conducted in an adjacent room 10 min after the dermoscopy skills training lecture finished.

### Sample size calculation

Informal preliminary investigations were first undertaken by S.F. with 16 colleagues with intermediate-to-advanced dermoscopy skills. Based on the results, we estimated that a sample size consisting of paired data for 52 participants (scores achieved using enhanced vs. standard images) should be sufficient to achieve a power of 80% and a significance level of 5% (two-sided) to detect an effect size of 0.4 between pairs (reasonably small effect size).

### Image-enhancement software

**Figure 1:**
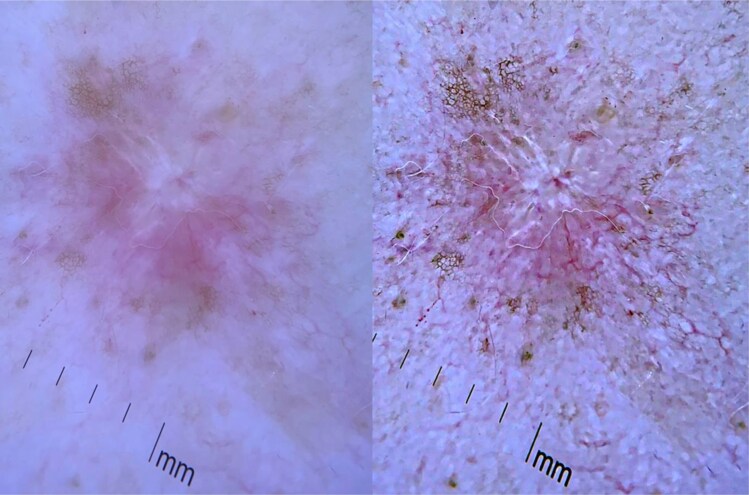
Dermatofibroma (benign lesion). Left: standard dermoscopic image. Right: enhanced image showing polarized specific white lines, vasculature and the pigment network more clearly.

**Figure 2:**
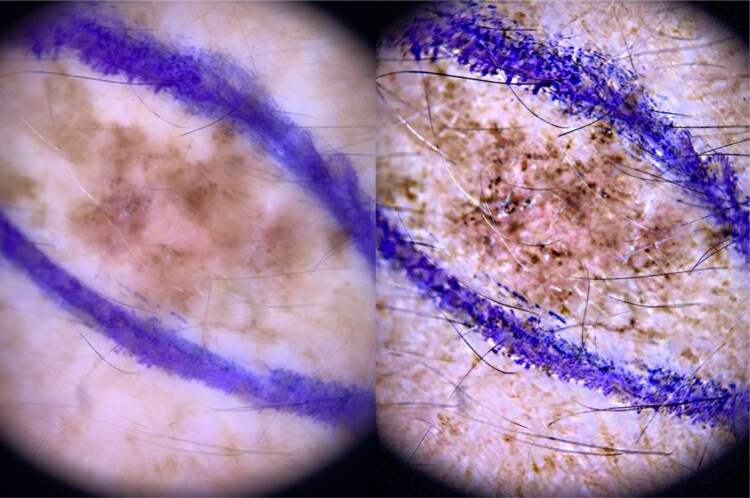
Melanoma *in situ*. Left: standard dermoscopic image. Right: enhanced image showing the irregular pigment distribution more clearly. Although it is unconventional to show pen markings, the sharpening of the pen lines helps demonstrate the enhancement effect.

**Figure 3:**
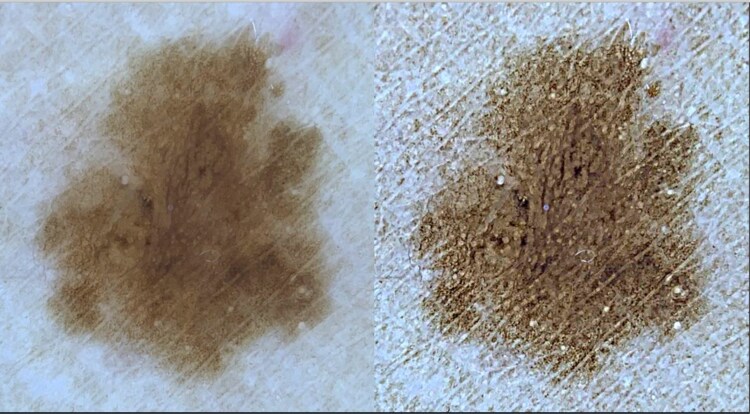
Benign lentigo. Left: standard dermoscopic image. Right: enhanced image. Although symmetrical, the pigment can appear more striking, with the potential for false-positive results.

The image-enhancement software was developed to enhance CM features, including asymmetry, atypical pigment network, blue–grey colouration, polymorphous vessels and polarized specific white lines, without altering the fundamental colours or structures within the dermoscopic images (Figures 1–3). A software prototype was made available for use by the authors.

### Data collection

The students who attended data collection were instructed to open the study materials on their personal smartphone via a QR code. Following an introductory segment during which J.H. guided participants through three example lesions, the students were asked to examine the standard dermoscopy image of the first lesion in the test set on their phone (zoom function was available). They were then asked to answer three dichotomous questions about the presence (score = 1) or absence (score = 0) of (i) lesion asymmetry, (ii) blue–grey colour and (iii) an atypical pigment network. They then used the 3-point checklist score to arrive at their diagnosis (scores ≤1 benign; scores ≥2 MIS/CM) for the standard image and then the enhanced image of the same lesion. For each case, participants were asked to rate how the enhanced image impacted their confidence in decision making.

The process was repeated for all 10 lesions in the test set. Feedback was provided by J.H. to the participant group between each lesion about the presence or absence of features from the three-point checklist, along with the lesion’s histological diagnosis. There was also an opportunity at the end of the test set for participants to provide subjective feedback. Student responses were autopopulated in an online Microsoft Excel spreadsheet.

### Statistical analysis

Numerical data were summarized as mean (SD) or median (interquartile range). Total student performance scores (maximum 40) were compared overall by image type (enhanced vs. standard) and also by lesion type (benign vs. MIS/CM) using the Student’s *t*-test. anova was performed to examine the effect of image enhancement on total student performance scores separately by lesion type, within the subset of seven MIS/CM lesions (maximum score 28) and then within the subset of three benign lesions (maximum score 12).

Incidence rate ratios (IRRs) with 95% confidence intervals (CIs) were calculated using Poisson regression models to assess the likelihood of correctly diagnosing the lesion type (melanoma vs. benign) based on image type (enhanced vs. standard). The model included lesion type, image type and their interaction term to examine whether the effect of image type on diagnostic accuracy differed for melanoma. The primary outcome was the number of correct diagnoses, with IRRs indicating the relative likelihood of correct readings.

Lastly, the numbers of true-positive, true-negative, false-­positive and false-negative responses achieved were used to determine the overall sensitivity and specificity. The diagnostic odds ratio (OR; the ratio of the odds of positivity in patients with disease relative to the odds in patients without disease) was also calculated, and the ORs of the random effects model are ­presented together with 95% CIs.

All statistical analyses were performed in R version 4.0.3 (R Foundation for Statistical Computing, Vienna, Austria). Statistical significance was assessed at a *P*-value threshold of <0.05.

## Results

**Figure 4:**
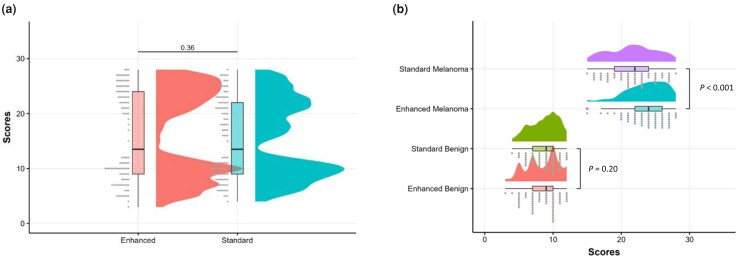
(a) Distribution of total student performance scores (maximum score 40) for the test set of 10 lesions. Comparison of diagnosis scores achieved using standard dermoscopy images compared with enhanced dermoscopy images. (b) Comparison of diagnosis scores for benign lesions (maximum score 12) and melanoma lesions (maximum score 28) under enhanced and standard imaging.

Of the 196 third-year students invited to participate via group message, 177 attended lectures on the day of testing and 61 (34.5%) agreed to stay behind to trial the test set. Total student performance scores did not differ significantly between standard and enhanced dermoscopy images for the 10-image set [mean (SD) 15.2 (7.1) vs. 16.1 (8.1); *P* = 0.36] (Figure 4a). However, with subanalysis of benign and melanoma lesions across both image types, melanoma lesions consistently scored higher (Figure 4b), and image enhancement resulted in a significant improvement in score. For benign lesions, the mean (SD) score was 8.39 (2.33) for enhanced images and 8.77 (2.05) for standard images. In contrast, melanoma lesions scored a mean (SD) of 23.7 (2.85) for enhanced images and 21.7 (3.42) for standard images (*P* < 0.001).

On subanalysis of the components of the 3-point checklist (Table 1), there was no significant difference in scores between enhanced and standard images in the asymmetry category. However, scores for atypical pigment networks (mean 5.87 vs. 5.05, difference of 0.82; *P* = 0.003) and blue–grey colour (mean 5.48 vs. 4.89, difference of 0.59; *P* = 0.01) were higher in the enhanced images of MIS/CM. Consequently, students achieved significantly higher overall scores diagnosing MIS/CM using enhanced vs. standard images (mean 6.11 vs. 5.52, difference of 0.59; *P* = 0.004).

**Table 1:** Differences in mean scores for the individual components of the 3-point checklist for cutaneous melanoma (asymmetry, colour, atypical pigment network) and the clinical diagnosis achieved by novice dermoscopists using enhanced dermoscopy images compared with standard dermoscopy images (maximum score 7 in each category)

Characteristic	Enhanced	Standard	Difference (95% CI)	*P*-value
Asymmetry	6.26	6.20	0.07 (−0.24 to 0.38)	0.68
Blue or grey colour	5.48	4.89	0.59 (0.14 to 1.04)	0.01
Atypical pigment network	5.87	5.05	0.82 (0.29 to 1.35)	0.003
Correct diagnosis	6.11	5.52	0.59 (0.20 to 0.98)	0.004

CI, confidence interval.

As shown in Table 2, there were no significant differences in overall diagnostic performance between standard and enhanced images, with an IRR of 1.04 (95% CI 0.93–1.18; *P* = 0.48). However, melanoma lesions were significantly more likely to be correctly diagnosed than benign lesions, with an IRR of 1.21 (95% CI 1.10–1.34; *P* < 0.001). The interaction between image type and lesion type suggested a lower diagnostic accuracy for melanoma lesions when using standard images compared with enhanced images, with an IRR of 0.87 (95% CI 0.76–1.01), although there was only weak evidence of an association (*P* = 0.06).

**Table 2:** Incidence rate ratios (IRRs) with 95% confidence intervals (CIs) and *P*-values for the likelihood of a correct diagnosis based on image type (standard vs. enhanced) and lesion type (benign vs. melanoma)

Characteristic	IRR (95% CI)	*P*-value
Image		
Enhanced		
Standard	1.04 (0.93–1.18)	0.48
Types		
Benign		
Melanoma	1.21 (1.10–1.34)	<0.001
Image × types (interaction term)		
Standard × melanoma	0.87 (0.76–1.01)	0.06

**Figure 5:**
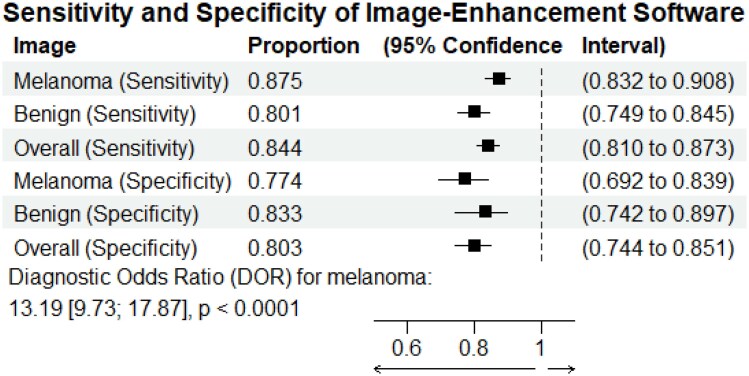
Sensitivity and specificity of the image-enhancement software.

The overall sensitivity of this image-enhancement software in helping this sample of novice dermoscopists to correctly diagnose early melanoma was 0.84 (95% CI 0.81–0.87), and the overall specificity was 0.80 (95% CI 0.74–0.85) (Figure 5). The diagnostic OR indicated that the odds of these students obtaining a correct diagnosis when observing histologically confirmed MIS/CM was 13.2 times higher than the odds of them obtaining a positive diagnosis when observing benign lesions (OR 13.2, 95% CI 9.73–17.9; *P* < 0.001).

## Discussion

Our study examined the utility of image-enhancement software on dermoscopy images to aid the early detection of CM. The software demonstrates potential as an accessible and inexpensive tool for enhancing recognition of CM. Third-year undergraduate medical students were able to identify atypical network and blue–grey colour in the seven MIS/CM lesions in our test set at a significantly higher rate than in standard images. Consequently, their ability to diagnose melanoma also improved. The students also reported a subjective improvement in diagnostic confidence when using the enhanced images.

The enhancements did not improve scoring on identification of lesion symmetry. We postulate that humans have an innate ability to identify symmetry, and this was the feature students scored best on for original and enhanced lesions.

The digital enhancements did not improve the accuracy of diagnosis for the benign lesions. Symmetry and the absence of melanoma-specific features are the main clues that a lesion is benign. Other than confirming a symmetrical distribution of pigment, it is not possible to digitally enhance the absence of a melanoma-specific feature. Despite the enhancements often making the lesions look more dramatic, the number of false-positive results did not increase.

Improvement in the diagnosis of melanoma lesions suggests the image-enhancement software shows sufficient promise to be studied further in terms of different groups of clinicians and types of lesions. As novice dermoscopists the medical students required some dermoscopy training prior to completing our test set; this would not be a requirement when working with more experienced dermoscopists. Alternatively, studies could be designed such that any percentage improvement from image enhancement could be determined separately from teaching benefit.

Polymorphic vessels are an important criterion for identifying ­difficult-to-diagnose CMs such as hypomelanotic melanoma.^16^ Although the 3-point checklist did not require the students to look at vessel features when reviewing our test set, our experience from piloting this tool with expert and intermediate dermoscopists is that the enhanced images are useful for highlighting blood vessels. The ability of image-enhancement software to enhance vessels has been noted previously.^12,13^ Vessel enhancement is an important part of existing Sparca software for assessing anterior eye conditions. We plan to explore this feature when trialling with more experienced dermoscopists identifying hypomelanotic melanoma, and will make the software available for study in clinical settings.

If the enhanced dermoscopy images continue to prove useful for education or clinical decision making, then the tools could be made widely and cheaply available in the form of a mobile phone app for use in a primary care or remote setting. A further possibility is for the software to be incorporated into an existing dermoscopy image management system.

## Data Availability

The data underlying this article are stored on the Research Data James Cook University platform and can be shared on request to the corresponding author.

## References

[1] Arnold M, Singh D, Laversanne M et al Global burden of cutaneous melanoma in 2020 and projections to 2040. JAMA Dermatol 2022; 158:495–503. 35353115 10.1001/jamadermatol.2022.0160 PMC8968696

[2] MelNet . New Zealand Melanoma Clinical Guidelines. Available at: https://melnet.org.nz/clinical-resources-and-research/quality-statements (last accessed 26 February 2026).

[3] Melanoma Institute Australia . State of the nation – a report into melanoma. Available at: https://melanoma.org.au/news/state-of-the-nation (last accessed 26 February 2026).

[4] Vestergaard ME, Macaskill P, Holt PE, Menzies SW. Dermoscopy compared with naked eye examination for the diagnosis of primary melanoma: a meta-analysis of studies performed in a clinical setting. Br J Dermatol 2008; 159:669–76. 18616769 10.1111/j.1365-2133.2008.08713.x

[5] Marghoob A, Braun R, Braun RP. An Atlas of Dermoscopy, 2nd edn. London: CRC Press, 2013.

[6] Skvara H, Teban L, Fiebiger M et al Limitations of dermoscopy in the recognition of melanoma. Arch Dermatol 2005; 141:155–60. 15724011 10.1001/archderm.141.2.155

[7] Rutjes C, Torrano J, Soyer HP. A 3D total-body photography research network: the Australian experiment. Hautarzt 2022; 73:236–40. 35029695 10.1007/s00105-021-04938-7 PMC8888503

[8] Horsham C, O’Hara M, Sanjida S et al The experience of 3D total-body photography to monitor nevi: results from an Australian general population-based cohort study. JMIR Dermatol 2022; 5:e37034. 37632874 10.2196/37034 PMC10334884

[9] Winkelmann RR, Farberg AS, Tucker N et al Enhancement of international dermatologists’ pigmented skin lesion biopsy decisions following dermoscopy with subsequent integration of multispectral digital skin lesion analysis. J Clin Aesthet Dermatol 2016; 9:53–5. 27672411 PMC5023003

[10] Pellacani G, Argenziano G. New insights from non-invasive imaging: from prospection of skin photodamages to training with mobile application. J Eur Acad Dermatol Venereol 2022; 36:38–50. 35738810 10.1111/jdv.18197 PMC9328152

[11] Braun RP, Marghoob A. High-dynamic-range dermoscopy imaging and diagnosis of hypopigmented skin cancers. JAMA Dermatol 2015; 151:456–7. 25535875 10.1001/jamadermatol.2014.4714

[12] Ayhan E, Toprak SF. Enhancing the appearance of vessels in dermoscopic images via a mobile photo editor application. J Am Acad Dermatol 2020; 82:e11–12. 31078604 10.1016/j.jaad.2019.05.008

[13] Blum A, Ellwanger U, Luedtke H. Features Amplifying Dermoscopy (FAD) for better evaluation in difficult pigmented and non-pigmented melanocytic skin tumors. J Dtsch Dermatol Ges 2014; 12:77–9. 24119234 10.1111/ddg.12202

[14] Soyer HP, Argenziano G, Farro P et al Three-point checklist of dermoscopy: a new screening method for early detection of melanoma. Dermatology (Basel) 2004; 208:27–31. 10.1159/000075042 14730233

[15] Zalaudek I, Argenziano G, Soyer HP et al Three-point checklist of dermoscopy: an open internet study. Br J Dermatol 2006; 154:431–7. 16445771 10.1111/j.1365-2133.2005.06983.x

[16] Zalaudek I, Kreusch J, Giacomel J et al How to diagnose nonpigmented skin tumors: a review of vascular structures seen with dermoscopy: part I. Melanocytic skin tumors. J Am Acad Dermatol 2010; 63:361–74. 20708469 10.1016/j.jaad.2009.11.698

